# Lenacapavir for HIV Prevention: A Commitment to Equitable Access and Partnership by Gilead Sciences

**DOI:** 10.1093/cid/ciaf116

**Published:** 2025-04-09

**Authors:** Jared M Baeten

**Affiliations:** Gilead Sciences, Foster City, California, USA

**Keywords:** HIV, PrEP, lenacapavir, prevention, access

## Abstract

Lenacapavir, a first-in-class HIV capsid inhibitor, is under development by Gilead Sciences for human immunodeficiency virus (HIV) prevention as pre-exposure prophylaxis (PrEP). This article responds to inquiries into Gilead's access efforts for low- and lower-middle-income countries and reviews the science behind lenacapavir and its investigational use for HIV prevention, the current state of Gilead's planning for equitable access, and the necessity for partnership to end the HIV epidemic. Clinical trial results are a culmination of years of dedicated science, and they are just the beginning; we look forward to sharing updates as milestones are reached. Gilead is committed to working with scientists, clinicians, communities, advocates, governments, and other stakeholders to achieve broad, sustainable global access to lenacapavir for HIV prevention, if approved. Lenacapavir holds potential for helping to end new HIV infections around the world, a potential that reflects the vision we have at Gilead: to end the HIV epidemic for everyone, everywhere.

## Teaser

This article reviews the investigational use of lenacapavir for HIV prevention, the current state of planning for equitable access by Gilead Sciences, and the ecosystem of partnership with scientists, clinicians, communities, advocates, and governments aiming to achieve broad, sustainable global impact.

**(See the Viewpoints by Hill et al on pages 547–54.)**

In June and September 2024, 2 phase 3 clinical trials released results after interim analyses met prespecified efficacy criteria evaluating twice-yearly subcutaneous lenacapavir, a first-in-class, highly potent, multistage inhibitor of human immunodeficiency virus (HIV) capsid, compared with background HIV incidence and once-daily oral Truvada (emtricitabine 200 mg and tenofovir disoproxil fumarate [F/TDF] 300 mg) for the investigational use as HIV pre-exposure prophylaxis (PrEP) [1, 2]. The 2 trials—PURPOSE 1 and 2, respectively—were global collaborations, with more than 100 clinical trial sites and hundreds of collaborating investigators in Argentina, Brazil, Mexico, Peru, South Africa, Thailand, Uganda, and the United States. The results of these trials were presented to scientific and consumer press, advocates, scientists, and policymakers around the world [3–5].

Despite the promise of HIV treatment and PrEP to reduce new infections, the world has recognized that scientific data on their own will not end the epidemic: access and uptake are essential for impact. Gilead Sciences is the innovator of lenacapavir and the regulatory sponsor of the PURPOSE trials. Gilead's history in HIV is one of scientific innovation coupled with a deep commitment to access around the world, all done in partnership with scientists, clinicians, communities, advocates, governments, and other stakeholders. This article discusses the science behind lenacapavir for HIV prevention, our current state of planning that aims at equitable access if it is approved, and the opportunity for partnership to achieve global impact.

## THE DEVELOPMENT OF LENACAPAVIR FOR HIV PREVENTION

**Figure 1.**
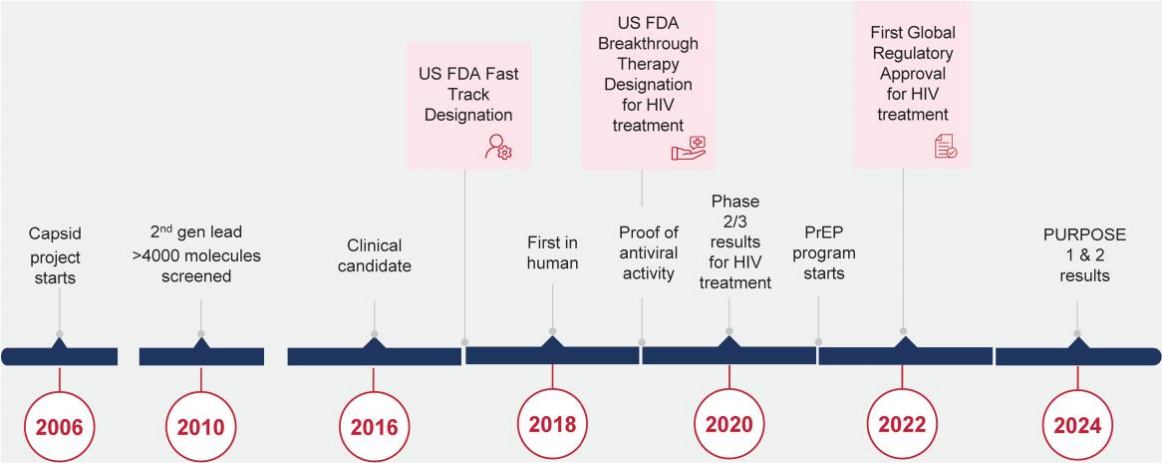
The development of lenacapavir for HIV prevention. Abbreviations: FDA, Food and Drug Administration; gen, generation; HIV, human immunodeficiency virus; PrEP, pre-exposure prophylaxis.

Nearly 2 decades of science preceded the PURPOSE 1 and 2 results (Figure 1). Beginning in 2006, and for a dozen years before entering first-in-human studies, more than 4000 molecules were synthesized and screened for activity against the HIV capsid by Gilead scientists, optimizing for potency and long half-life [6]. Lenacapavir for the treatment of multi-class–resistant HIV in highly treatment-experienced adults, in combination with other antiretroviral medicine(s), was first approved in 2022; in locations where it was not or has not yet been approved, we have provided it free of charge through compassionate-use programs to individuals on 6 continents through Gilead's managed-access program [7].

**Figure 2.**
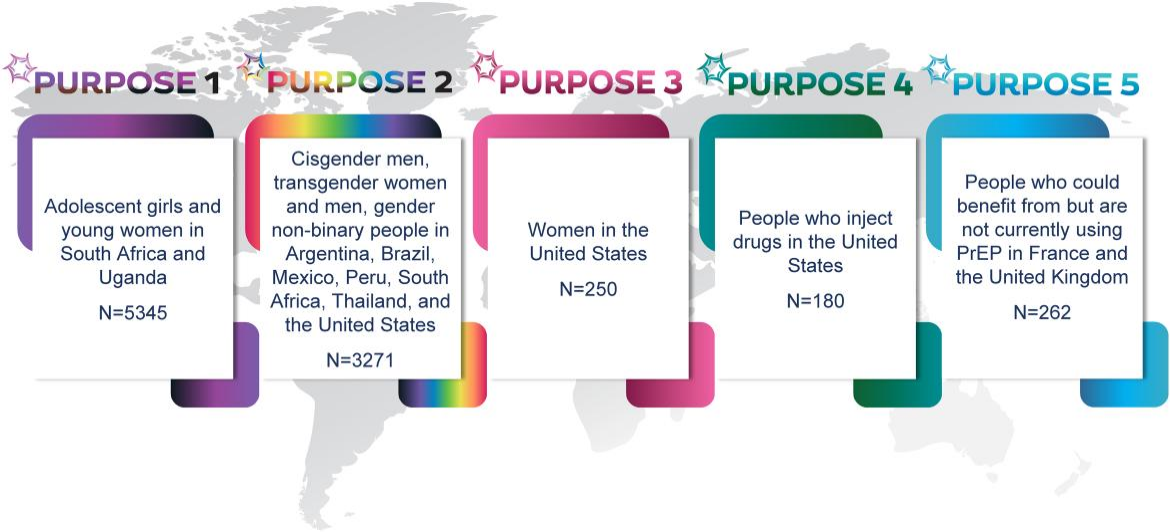
PURPOSE program: lenacapavir for PrEP. Abbreviation: PrEP, pre-exposure prophylaxis.

The innovations in science within the design of the PURPOSE program include a novel primary endpoint; assessing measured HIV incidence against an expected background incidence; and a strategy developed in collaboration with scientists, community members, regulatory agencies, and pharmaceutical companies for the advancement of novel PrEP agents [8]. PURPOSE 1 included adolescents and pregnant and lactating women, data that are particularly important for low- and lower-middle-income countries, where young women account for a disproportionate fraction of new HIV infections and have had generally low uptake and use of PrEP. PURPOSE 2 included, for the first time in a phase 3 PrEP program, transgender men and gender nonbinary individuals [9]. Ongoing trials (PURPOSE 3, 4, and 5) complement the pivotal trials with additional populations (Figure 2). The PURPOSE program, operating across 5 continents and across geography, race, ethnicity, gender, and age, is the most broadly representative clinical trial program ever conducted for HIV prevention. *Science* magazine recently named lenacapavir its 2024 breakthrough of the year [10].

The scientific innovation within the PURPOSE program is a result of intentional activities to engage and listen to communities, advocates, clinicians, scientists, and policymakers on the evolving needs of people who might need or want PrEP. Standing Global Community Advisory Groups—with representatives of the communities in the trials—engaged with the study teams from before protocols were initiated through trial results. Gilead closely engaged multiple regulatory agencies to ensure that the clinical trial program would be both rigorous like prior PrEP trials and able to address questions never addressed before in a PrEP program. Importantly, global access planning was anticipated early. For example, as part of the regulatory review of the PURPOSE 1 study in South Africa and Uganda, Gilead committed to post-trial drug access: both intention to register in those countries if lenacapavir had evidence from the trial of safety and efficacy and bridging supply for trial participants until potential approval locally. Today, participants in the PURPOSE 1 and 2 trials are being offered open-label lenacapavir and will have access assured through Gilead until potential regulatory approval and availability in their countries.

The PURPOSE program has learned from 2 decades of work on PrEP by researchers from countries around the world that collaborative, equitable science must engage community, adhere to the Good Participatory Practice Guidelines, build capacity, and establish readiness [11–13]. Since the first regulatory approval of PrEP in 2012, more individuals have started PrEP in low- and lower-middle-income countries than in high-income countries [14], a testament to the importance of providing access. Nevertheless, more than 1.3 million new infections still occur each year [15], emphasizing how the development of and access to additional PrEP options are needed to help end the epidemic.

## INNOVATIONS IN EQUITABLE ACCESS: PRIOR HISTORY AND FUTURE PROSPECTS

Gilead's commitment to innovation has never been limited to drug development. Beginning in 2003, Gilead was the first company to enable generic manufacturers to produce its medicines at large scale for low- and lower-middle-income countries ahead of patent expirations through direct voluntary licensing [16]. Gilead was also the first pharmaceutical company to support the Medicines Patent Pool (MPP), a public health organization that works to increase access to medicines for low- and middle-income countries, with more antiretroviral agents licensed to the MPP from Gilead than from any other company [17]. Today, Gilead innovations—for HIV and also viral hepatitis and coronavirus disease 2019 (COVID-19)—are licensed to generic manufacturers in more than 100 low- and lower-middle-income countries. In 2023 alone, more than 20 million HIV and hepatitis B treatments based on Gilead therapeutics were made available to people living in these countries through generic manufacturers. For COVID-19, direct voluntary licenses to 9 generic manufacturers for remdesivir (Veklury), issued effectively simultaneously with US Food and Drug Administration (FDA) approval [18], resulted in 8 million treatments between 2021 and today. In addition to generic manufacturer partners, in 2023, more than 11 million units of Gilead-branded medicines were delivered to nearly 250 000 individuals in low- and lower-middle-income countries.

Beyond manufacturing, Gilead is committed to continued scientific innovation to provide solutions for the evolving needs of people affected by HIV around the world. Through partnerships, collaborations, and charitable giving, the company also aims to improve education, expand access, and address barriers to care. To that end, Gilead has been repeatedly recognized, with the Gates Foundation, as 1 of the top 2 leading philanthropic funders of HIV-related programs in a report released by Funders Concerned About AIDS [19].

Innovations in health can take decades to reach low-income countries, which are often where the need is greatest [20]. To shift this paradigm, Gilead began detailed planning for global access for lenacapavir 2 years ahead of the results of PURPOSE 1 and PURPOSE 2. Our access strategy for high-incidence, resource-limited countries, which are primarily low- and lower-middle-income countries, reflects extensive consultations with HIV-affected communities worldwide as well as governments, advocates, multilateral organizations, individuals who need or want PrEP, and community partners. We have received extensive and wide-ranging advice—some broad, some very specific, and some contradictory. We have made decisions guided first and foremost by what we anticipate would be best for people who want or need PrEP in low- and lower-middle-income countries and an intent for our innovation in equitable access to match the innovation that has been lenacapavir science. Our planning for low- and lower-middle-income countries is guided by 4 priorities: delivering lenacapavir with speed, at sufficient volume to meet demand, at prices that enable widespread availability, and in partnership in a multisector ecosystem, if it is approved.

### Speed

Gilead initiated a series of global regulatory filings at the end 2024 that will continue through 2025, with near simultaneous filings across global settings with the highest HIV incidence [21, 22]. This approach differs substantially from that used for most pharmaceutical products, which are filed and receive approval first in high-income countries and then, over a period of years, are included in filings for middle- and finally low-income settings. In October 2024, the US FDA granted lenacapavir for PrEP Breakthrough Therapy Designation, which is intended to expedite the development and review of new drugs that may demonstrate substantial improvement over available therapy, and also granted a rolling review for lenacapavir for PrEP, a process that allows a company to submit sections of the application for review as they are completed.

We are pursuing frameworks intended to facilitate faster access in key low-income countries, including the European Medicines Agency's (EMA's) EU Medicines for All, the World Health Organization's (WHO's) collaborative review and prequalification procedures [23]. EU Medicines for All provides a technical opinion on medicines intended for use outside of the European Union and rapid availability of WHO prequalification, with review performed collaboratively by the EMA, WHO, and scientific experts from regulatory authorities from low- and lower-middle-income countries. The procedure can run in parallel with a European centralized filing, and thus we believe the result could enable Gilead to secure approvals in key high-incidence, resource-limited countries quickly after a European Union approval. While we control the timing of submission to national regulatory agencies, the average timeline of review by national agencies can range from 6 months to 2 years or more. Updates on regulatory filings for lenacapavir for PrEP will be shared publicly as discussions with regulatory bodies progress.

Within weeks of the PURPOSE 2 results, Gilead signed nonexclusive direct voluntary licensing agreements with 6 pharmaceutical companies with the capacity to synthesize, manufacture, and supply high-quality, low-cost versions of lenacapavir for 120 mainly low- and lower-middle-income countries [24]. To our knowledge, this is the earliest and geographically broadest voluntary licensing strategy ever developed for an anti-HIV agent and reflected more than 1 year of technical review of potential generic manufacturers. Direct voluntary licenses enable Gilead to initiate technology transfer quickly and problem-solve with the generic manufacturers, accelerating their readiness; to that end, technology transfer to the 6 manufacturers has already been completed. Gilead's licensing contracts include not only lenacapavir for PrEP but also lenacapavir for the treatment of HIV in heavily treatment-experienced persons with multi-class–resistant HIV infection. The licensees are based in Africa and Asia, and the number of companies was chosen to ensure sufficient supply and competition for high-quality, low-cost product. These contracts are royalty-free, meaning that generic manufacturers will pay nothing to Gilead.

### Volume

It will take time for voluntary licensees to build manufacturing capacity for lenacapavir. Thus, Gilead has committed to providing Gilead-manufactured lenacapavir, in collaboration with procurement and distribution partners, to low- and lower-middle-income countries to bridge the gap until licensed generic versions are available. Gilead has consulted extensively and remains in active dialogue with the key procurement agencies to understand product demand in low- and lower-middle-income countries in the period during which we would supply lenacapavir. To that end, we recently contracted at-risk manufacturing capacity to produce vials of lenacapavir and corresponding initiation tablets for these countries, enough supply for approximately 2 million or more individuals over the time before generic entry. This Gilead-manufactured supply for low- and lower-middle-income countries will be ring-fenced so it cannot be diverted away from these countries to higher-income countries. Independent groups have estimated that the period while generic manufacturers are ramping up their technical and manufacturing capacity could be 2 years or less, considerably faster than prior PrEP products [25]. Achieving those timelines will require generic manufacturers to synthesize and package lenacapavir, conduct bioequivalence studies to demonstrate comparability to the originator product, and file their own regulatory packages with countries; we are working to assist generic manufacturers in these activities. The lenacapavir molecule is synthesized through a complex, multistep process and the final product requires sterile vial filling; generic manufacturers will need to ensure end-to-end quality.

### Price

For high-incidence, resource-limited countries, we plan to price lenacapavir for PrEP at no profit to Gilead to ensure availability until generic licensees come online. While regulatory reviews are ongoing, we are working with our partners around the world to optimize costs of lenacapavir for PrEP for these settings and, in turn, determine access pricing for Gilead-manufactured lenacapavir. We will stay true to the company's history and commitment to enabling broad access, while incentivizing generic manufacturers to enter the market and supply medicines at progressively lower prices over time, as has been demonstrated with the history of oral PrEP in low- and lower-middle-income countries [26].

For upper-middle and high-income countries, Gilead is actively working on multiple ways to support access. For example, in Latin America, where PURPOSE 2 in part was conducted, approximately 300 000 people take Gilead-produced medications for HIV each day, and we have on-the-ground teams and offices across the region. Planning for these countries, incorporating input from advocates and global health organizations, is ongoing, including for the PURPOSE 2 countries of Argentina, Brazil, Mexico, and Peru. We are exploring several innovative strategies to support access to lenacapavir for PrEP for upper-middle-income countries, including tiered pricing, and we are working to establish fast, efficient pathways such as timely regulatory filings, planning for manufacturing at volumes that would meet demand and manufacturing infrastructure planning, discussions with governments, and leveraging partnerships for the long-term that will build and sustain impact. To be clear, the US list price for lenacapavir for the treatment of persons with multi-class–resistant HIV will not be the reference point for lenacapavir for PrEP pricing, for any country.

### Partnership

Gilead's access strategy for low- and lower-middle-income countries has been informed by hundreds of hours of listening to and engaging with more than 100 global health advocates and organizations. Those discussions shape our work daily, and they remind us that Gilead cannot do this work alone. An ecosystem of partnership will be needed [27], with all partners leaning in, collaborating and coordinating to achieve access and availability of lenacapavir, if approved, in low- and lower-middle-income countries. We have been actively and publicly engaging with partners from governments and nongovernmental organizations to commit to work that aims at achieving timely equitable access and impact [28–30]. Global procurers, such as the US President's Emergency Plan for AIDS Relief (PEPFAR) and the Global Fund, will need to secure agreements with generic manufacturers (and with Gilead until those are operating at scale) to buy sufficient volume to help achieve the potential impact we all seek. Global normative agencies, such as the WHO, must execute guidelines and prequalification to facilitate country guidelines and procurement. National governments around the world must develop plans for HIV prevention, including PrEP; must finance HIV prevention; and must enable their regulatory authorities to conduct their rigorous reviews. Generic manufacturers must move with speed to produce, register, and distribute high-quality lenacapavir at a large scale and at a sustainable price.

## GOING FORWARD IN PARTNERSHIP

Innovations only realize their full potential if they reach people, and access can be achieved only through partnerships among scientists, clinicians, governments, normative agencies, communities, and advocates from around the world. The results of the PURPOSE 1 and 2 trials are a culmination of years of dedicated science, and they are just the beginning of years of work to be done, in partnership, to achieve the potential impact that lenacapavir could have.

I have dedicated my career, in academia and now at Gilead, to ending HIV globally [31]. I have worked on PrEP and the use of antiretrovirals for prevention from pivotal clinical trials to implementation science to delivery and impact at scale [11, 32–37]. In partnership with investigators from low- and lower-middle-income countries, I have led work that resulted in the first medication approved as PrEP [11], the first long-acting PrEP option [38], and now lenacapavir. I am committed to ensuring that PrEP contributes to ending HIV, and, in this article, I represent thousands of Gilead colleagues who also work every day towards a goal of one day not having to work on HIV at all. At Gilead, we are scientists, physicians, public health professionals, patients, and advocates. We are proud of the innovation in medicinal chemistry, preclinical and clinical science, trial design and inclusion, and global collaboration that is behind lenacapavir, the PURPOSE trials, and access planning.

Gilead is committed to ensuring broad, sustainable global access to lenacapavir for PrEP, if approved. This commitment is guiding every step of our strategy planning. We are applying learnings from our decades of innovation and leadership in global access to medicines. We thank the people and organizations who have provided counsel on our lenacapavir for PrEP access strategy and who are partnering already towards solutions. We look forward to sharing further updates as milestones are reached. We recognize the potential that lenacapavir could hold for helping end new HIV infections around the world, a potential that reflects the vision we have at Gilead: to end the HIV epidemic for everyone, everywhere.

## Note

***Financial support.*** This research was supported by Gilead Sciences.
